# An Extended Home Visit Programme Within the Swedish Child Healthcare System for First-Time Parents in Scania, Sweden: A Study Protocol

**DOI:** 10.3389/fpubh.2021.537468

**Published:** 2021-02-09

**Authors:** Elisabeth Mangrio, Lisa Hellström, Eva-Lotta Nilsson, Anna-Karin Ivert

**Affiliations:** ^1^Department of Care Science, Faculty of Health and Society, Malmö University, Malmö, Sweden; ^2^Department of School Development and Leadership, Faculty of Learning and Society, Malmö University, Malmö, Sweden; ^3^Department of Criminology, Faculty of Health and Society, Malmö University, Malmö, Sweden

**Keywords:** child-health care, early life, health-professionals, health outcome, home-visits

## Abstract

**Background:** The Swedish Child Healthcare (CHC) system aims to provide equal and fair health care for all children and families in Sweden. Currently in Sweden, the CHC offers every family two home visits during the child's 1st year of life. During 2019, an extended home visit programme, called Grow Safely, was started in the region of Scania for first-time parents. The aim of the extended home visit programme was to provide support for first-time parents in order to improve the overall health of the child and family and contribute to better conditions for equal health. Instead of two home visits during the 1st year, a subsample of first-time parents would receive six visits during the child's first 15 months. These six visits would be conducted by CHC nurses and social workers, midwives, and dental assistants. In the present paper, we describe a research project related to the regional extended home visit programme; the project aims to illuminate the experiences of the participants and to investigate the perceived benefits of the programme in relation to improved health, social and emotional interaction between parent and child, and attitudes toward authorities and surrounding society.

**Method/Analysis:** In order to evaluate the introduction of the intervention, three qualitative interview studies and one quantitative study with follow-up questionnaires will be conducted. Since the research project also comprises studies focusing on the implementation and expectations of politicians, civil servants, organizational managers, and professionals working within the programme, interviews within these fields will be conducted.

**Discussion:** Sweden has a well-established CHC programme, but improvements are always possible. Previous research has shown that home visits are an effective tool to improve both the child's physical and mental health as well as the parents' well-being. However, this kind of intervention involves a significant investment from all organizations involved in the home visits; hence, it is important that the intervention is evaluated. The research project described in the present paper intends to examine the impact of the intervention, and its findings will aid decisionmakers in determining the future of the home visit programme.

## Introduction

The Swedish population is considered in good overall health, but differences still exist within the population mainly due to socio-economic factors (1). There is ongoing work aimed at reducing health inequalities for the population as a whole through preventive work against diseases, which in turn will contribute to a sustainable society (2). This goal is well in line with the WHO's work with the Commission on Social Determinants of Health (3). A child's early life experiences have a great effect on, among other things, the child's learning, health, and behavior (4). Furthermore, studies indicate that early life experiences could have long-term effects and may affect a person's well-being and ability to function in adulthood; therefore, early life interventions could also have an economic impact on the societal level (5, 6). For example, oral health has been shown to benefit from early intervention in order to prevent caries, and introducing early tooth brushing has been shown to be an effective way of preventing caries (7). A child is most susceptible to environmental influences from the prenatal period up to the age of three. Therefore, investment in this period is one of the most efficient and effective ways to eliminate inequality in health (4, 5), which necessitates early preventive efforts targeting families that are in need (4).

In Sweden, an ambitious Child Health Care (CHC) programme is offered to all children, with a total of 17 appointments from birth to age five. During the child's 1st year, two of these appointments are carried out as home visits, and if there is a special need, extra home visits are offered (8). Studies focusing on home visits in Finland (9, 10) and the United States (11, 12) show positive effects for first-time parents and their children, both in the short and in the long run. The positive health effects for infants include improvements in both physical and mental health as well as decreased emotional sensitivity and fewer speech delays (9–12). Research shows that the practical guidance given during home visits is also beneficial for the parents (13). An American study of home visits that looked at families in socio-economically deprived areas during the child's first 2 years reveals that these visits prevented mortality among both parents and children (14). In addition, Landry et al. (15) showed that home visits that included or centered around play and learning strategies resulted in increased emotional and social interaction as well as increased responsiveness. A Swedish study of parents residing in Sweden but born outside the EU shows that the parents were grateful for the Swedish CHC and the home visits since they did not all receive this type of help in their home countries (16). In 2013, an extended home visit programme was introduced in a socio-economically deprived area in Sweden called Rinkeby. It focused on first-time parents and included six home visits during the child's first 15 months (17). The home visits were conducted by pediatric nurses and social workers, and a total of 119 families participated in the programme. An evaluation of the extended home visit programme in Rinkeby showed improved immunization coverage for measles, mumps, and rubella (MMR) as well as fewer ER visits (18). Findings from the evaluation also suggested that good collaboration between the pediatric nurses and social workers conducting the home visits increased trust among the parents toward these professions (18). Apart from the above-mentioned Swedish study (17), to the best of our knowledge, no other studies have been conducted that explicitly focus on the CHC collaborative home visits by pediatric nurses, midwives, social workers, and dental assistants.

Since earlier studies show the health benefits of home visits during the child's 1st year of life, both for the parents and the child (9–12), there is a need to continue to investigate this matter in a Swedish context. In 2019, the Skåne Regional Council started an extended home visit programme called Grow Safely, in the county of Scania (Skåne), Sweden. This programme is similar, but more extensive, to the one implemented in Rinkeby. This programme is a part of a greater governmental investment during 2019–2020 of SEK120 million for the Swedish CHC, with the goal of trying to achieve more equal health among the children, with a special focus and concern for socially deprived areas in Sweden (19). The county of Scania, where the programme will be carried out, comprises both rural and urban areas. A total of 1.3 million citizens reside here. Of this population, 21% were born outside of Sweden and most migrants come from the former Yugoslavia and the Middle East (20). In the county of Scania, there are approximately 148 CHC centers catering to around 100,000 children under the age of six (21). A report investigating health among children in Malmö (the largest city in Scania) reveals that there are some children who suffer from poor health, poor living conditions, and poor economy. A large proportion of these children have refugee backgrounds, which in itself increase their vulnerability (22). Another annual report on the child healthcare in the county of Scania reveals that 5% of 4-year old children do not attend the 4-year old screening, which includes motor and language development and a screening for hearing and vision (23). For children residing in socioeconomically deprived areas in Scania, 16% of 4-year old children miss out on these visits (23), which shows the importance of working toward the improvement of equal health among infants and children in Scania.

The project in Scania plans to increase home visits for first-time parents: Instead of two home visits during the 1st year, they would receive six home visits during the child's first 15 months. By increasing the number of home visits for first-time parents, the project aims to improve the overall health of the parents as well as the child. These six visits will be conducted by CHC nurses in collaboration with social workers, midwives, and dental assistants; the CHC nurse will conduct each home visit together with one of these professionals (see Table 1). Region Scania's motivation for initiating this programme was to provide support to contribute to accessibility to care and equal health within the population in Scania. This intervention differs from the ordinary programme in the extended amount of home visits and the cooperation between the professions that are a part of these visits, which does not exist in the regular programme. All the CHC centers in the county of Scania were invited to apply for participation in the extended programme. Four CHC centers volunteered to participate in the intervention in 2019, and in 2020, another 23 CHC centers were included. A total of 27 CHC centers out of 147 thus participated. The relative limited interest in the programme among CHC centers could be due to a lack of work resources and a lack of collaboration among authorities. The intervention period extends over 3 years (i.e., until the end of 2022). These CHC centers represent 18 municipalities within the county, comprise both rural and urban areas, and differ in relation to the number of migrants residing in the areas they serve. The participation of first-time parents is voluntary. A limited number of infants per CHC center will be enrolled in the extended programme; the remainder will be a part of the ordinary CHC programme and will have the option to participate in the comparison group of the current study. The number of first-time parents per CHC center that are enrolled in the programme depends on the capacity of each CHC center with respect to increased workload. Each CHC agreed and signed up to invite either 20 or 40 first-time parents into the programme. The selection of the first-time parents who would be offered participation in the extended home visit programme has been the responsibility of each respective CHC. The project leader of the research group participated in the group that planned the project, where, among other things, ethical aspects of this process have been discussed. However, the selection of participants was done by each CHC in collaboration with the planning group of the project. For example, some CHC centers invite parents that previously visited the midwives involved in the programme in order to maintain continuity of care. Some CHC centers chose to invite parents based on where they reside, with the aim of ensuring reasonable commute times for the health professionals conducting the home visits. Other CHC centers with a greater number of first-born infants chose to invite every first and second child born per week. To conclude, the selection process differs between CHCs. Regardless of this, the process has been documented carefully in collaboration with the planning team of the project.

**Table 1:** Program over visits at the child health care including home visits and type of research.

**Ordinary child health care program(Comparison group)**	**Extended home-visit program: Home-visit at what age and professions**	**Type of research**
1–2 weeks	1–2 weeks ¤ Home-visit by midwife and pediatric nurse	Interview with parents
Home-visit by pediatric nurse	3 weeks Home-visit by social worker and pediatric nurse	
2 months ¤	2 months ¤	Questionnaire for parents
	4 months ¤ Home-visit by social worker and pediatric nurse	Interview with parents
8 months	8 months ¤ Home-visit by dental assistant and pediatric nurse	Interview with parents
Home-visit by pediatric nurse	10 months Home-visit by social worker and pediatric nurse	
¤	15 months ¤ Home-visit by social worker and pediatric nurse	Questionnaire for parents

^¤^*Apart of the research*.

## Methods and Analysis: the Research Project

The research project that will evaluate this programme will be conducted by a multi-disciplinary and multi-professional research group from Malmö University, with researchers from the departments of Care Science, School Development, Public health, Criminology, Social Work, Sport Science, and Odontology. The research applies a mixed-methods approach, which includes both qualitative and quantitative data collection.

Qualitative studies will be carried out with first-time parents at three different stages of the programme, all of which will be conducted by the research team. First, one interview study will take place after the first home visit, which is conducted by a midwife and a pediatric nurse when the baby is 1 to 2 weeks old. The focus of this interview will be the parents' experience of the home visit, parenthood-related issues and the parents' current concerns, and the parents' perception of how well the home visits address these concerns. It will also cover aspects of the parents' perception of the collaboration between the professions involved. The second interview study will be carried out after the third home visit, which is conducted by a social worker and a pediatric nurse when the baby is 4 months old. This second interview will focus on the parents' experience of the home visit and the potential knowledge gained from the visit. It will also cover aspects of the parents' perception of the collaboration between the social worker and the pediatric nurse. The last interview study will take place after the dental assistant and pediatric nurse have carried out home visits, when the baby is 8 months old. In these interviews, the focus will again be on the parents' experience of the visit, the knowledge gained from the visit, and the parents' perception of the collaboration between the dental assistant and the pediatric nurse. All interviews will be carried out using a semi-structured interview guide that includes questions covering the parent's experiences, knowledge increase, and feelings of support, as well as their perceptions of receiving home visits by two different professions in collaboration. All eligible caregivers are invited to take part in the interview, and the interviews will be conducted within 3–4 weeks after the home visits described above. The interviews will be conducted by researchers within the research group. An authorized translator will translate the interview questions into a preferred language if needed. The parents from the first interview will be asked to participate in the subsequent interviews, but the parents have the right to decline at any point. That is, the research interviews do not intend to follow up on the same families, and new families will be recruited for each wave of interviews. The interview study has received ethical approval in Lund, Sweden (Registration no. 2018/841).

In order to investigate the perceived benefits of the programme in relation to improved health, social and emotional interaction between parent and child, and attitudes toward authorities and the surrounding society, a baseline questionnaire will be distributed to the parents when the child is 2 months old, and a follow-up questionnaire will be distributed when the child is 15 months old. The composition of these questionnaires is largely based on earlier studies done within the CHC in Scania (24) as well as the evaluation of the Rinkeby extended home visit programme (17). The questionnaires are to a great extent composed and based on previous research that has been tested for validity [(24); Lindberg et al., (submitted)]. The questions target areas such as sociodemographic factors, economic factors, and employment/parental leave, equal parenting, breastfeeding habits, health care access, physical activity, social interaction with the child, and contentment and trust in health care settings as well as other societal institutions.

For the quantitative part of the research project (the questionnaire study), a comparison group will be recruited among parents not enrolled in the extended home visit programme but participating in the ordinary CHC programme. Parents within the comparison group will be selected from the same CHC programme that is enrolled in the extended home visit programme Grow Safely. Parents not invited to Grow Safely or who decline participation, are asked to be invited to fill out the questionnaire as part of the comparison group. Given that the comparison group is recruited from the same CHC center means that there is greater possibility that they share sociodemographic characteristics with the intervention group. The comparison group will fill out the same questionnaire as the intervention group. It could be preferable to conduct a power calculation in order to estimate the number of first-time parents needed to be enrolled in both the intervention group and the comparison group. However, this is not done due to different reasons. This is a total survey design where all first-time parents that get invited to participate in either Grow Safely or the ordinary programme (comparison group) at each CHC center that is engaged in Grow Safely, are invited to participate in the survey. In addition, it is difficult to estimate the effect of this programme since we have several outcomes and the programme is conducted during a limited time and with limited economic resources to cover such costs.

In future research, the children enrolled in Grow Safely can be followed in, for example, health care registers, but this will require an additional ethical application and most likely informed consent from the families.

The parents in both groups will be able to fill in the questionnaire online, on paper, or over the telephone with the assistance of a researcher. If the parents are not able to understand Swedish, a translator will be booked, and the questionnaire will be carried out together with both a researcher and a translator. Both parents will be given the opportunity to fill in their own questionnaire.

In order to investigate the extended home visit programme's potential effect on the oral health of the children included in the study, the research project will observe the number of caries at the age of 3 years in both programme groups. This will be done by linking the participating children to a register that covers number of caries at the of age three. When the child reaches 2 months of age, the parents will be asked to provide their consent to link their children's data to that register. This investigation was subjected to ethical review (2019/03266).

In addition to the studies' focus on the experiences of the parents and the development of the child, the research project also comprises studies that focus on the implementation and expectations of politicians, civil servants, organizational managers, and professionals working within the programme. To examine the organizational perspective—namely, how the programme is implemented and what the incentives and expectations are—the study includes interviews with the politicians involved in the decision to implement the programme, including the chairman and the vice chairman of the Regional Council. To deepen the knowledge of the organization and planning of the programme, the study also includes interviews with officials responsible for implementation. Furthermore, the CHC nurses, social workers, midwives, and dental assistants involved in the implementation of the programme will be invited to participate in interviews focusing, for example, on their expectations for the implementation of the programme and their experiences of being involved in the programme and collaborating with practitioners from other professions. We plan to be able to interview around 10 health professionals from each stakeholder. The interview guide will cover questions that will capture the perception of the home visit and the collaboration with other health professionals, challenges and success factors in the collaboration within the programme, experiences of the counseling team sessions, and their experiences with visiting families in different social and challenging situations.

## Discussion

The extended home visit programme initiated by Region Skåne seeks to improve the overall health of first-time parents and their children in Scania, Sweden. The reduction of health-related inequalities is currently at the forefront of policy initiatives around the world, and the Marmot Review emphasizes the work of reducing inequalities regarding children's health (25). Marmot highlights the need for knowledge to be linked to action on policy objectives, such as giving every child a good start in life and strengthening the role and impact of ill-health prevention (25). Society can ensure every child has a good start in life by providing support to the parents to enable them to make the most of the opportunities they are given. The Swedish CHC already has a well-established programme (8), but improvements that allow the CHC to provide better support to the families are always possible. Research has shown that home visits are an effective tool to improve both children's physical and mental health and parents' well-being (9–12). Home visits are also an effective method to prevent ill health, which could reduce inequalities among children (25). Moreover, extended home visits, such as the added visits in the programme implemented in Scania, could provide an opportunity for different professionals to collaborate around the families at an early stage. Early social interventions have been proven effective for at risk families (5); therefore, it could be assumed that these families would greatly benefit if social workers come in contact with them at an early stage. The extended home visit programme has several ways of working with the social interaction between the child and the parents, and research shows that children are very susceptible to environmental influences *in utero* up to the age of three (4, 5). Including social workers for a portion of four of the home visits during the extended home visit programme could help reduce ill health at an early age and improve the interaction between the child and the parents. These benefits have long-term effects, since we know that early-life experiences heave the potential to affect the child's well-being later on in life (5, 6). Consequently, these interventions could have long-term physical, mental, and social effects on the societal level as well (5, 6).

Since the beginning of 2020, the world, Sweden included, has seen the effects of the coronavirus pandemic, and we can assume this has had an effect on the ongoing programme. The CHC centers have been open the whole year and have been able to continue their ongoing work with Grow Safely. When it comes to the research, the questionnaires have been able to be continually sent out since they are administered online. The interviews have been carried out with the families but have mainly been conducted by phone due to social distancing recommendations. The interviews focusing on the organization and among professions, have and will be conducted through Zoom.

## Ethical Considerations

This research project involves a number of methodological and ethical issues that should be mentioned. As the aim of the extended home visit programme was to reach both rural and urban areas, a high proportion of the participating first-time parents were likely born outside of Sweden. These parents might have only recently arrived in Sweden, be new to the Swedish language, or face both social and economic challenges (26). This means that we as researchers need to address the various ethical aspects related to these possibilities. This is done by providing information letters that are translated into 10 different languages and access to interpreters where needed.

The information letters will be provided by the CHC nurses before the researchers contact the families. These information letters include information about the study and clarify that the study is on a voluntary basis and participants can withdraw from the research at any time without consequences.

Further, challenges could arise in recruiting participants among the first-time parents who are either receiving extended home visits or getting the usual care programme. A possible barrier is that participation in interviews or filling in a questionnaire could require the participants to set aside a significant amount of time; on the other hand, they may see the benefit of being able to affect the outcome of the Swedish CHC in the future. Since the parents could be asked to both fill out a questionnaire and participate in interviews, and this could entail a significant time commitment, we chose to avoid asking parents to participate in all three interview studies whenever possible. However, if the parents had an interest in doing so, they were welcome to take part in all three. In addition, we may also face challenges as researchers since our research encompasses a wide geographical area. For instance, conducting interviews while coordinating any assistance needed to fill out questionnaires will be time-consuming. On the other hand, this greater geographical area gives a broader, more diverse picture since it covers both urban and rural areas of the county. And a good collaboration has been established with all CHC centers, which enables us to carry out our research.

## Concluding Remarks

The kind of intervention described above involves a significant investment from all organizations engaged in the home visits; hence, it is important that the intervention is evaluated. The research project described in the present paper intends to examine the introduction of the programme, and its findings will aid decision makers in determining the future of the home visit programme.

## Data Availability Statement

Pursuant to national legislation, ethical review boards in Sweden do not allow the release of sensitive raw data to the general public. Therefore, we cannot share our qualitative data. The quantitative data is not yet collected.

## Ethics Statement

In the current research project, both the qualitative studies and quantitative study have received ethical approval (Reg No.: 2018/841, Lund University, Sweden, and Reg No.: 2019/03266, Uppsala University, Sweden, respectively). Before the qualitative studies are conducted, the interviewees will fill in a participant consent form. For the quantitative study, the parents that fill in the questionnaire consent to participation by answering a question about their willingness to participate, which is a part of the questionnaire.

## Author Contributions

The first author EM took the lead role in writing the paper and the other three contributing authors LH, E-LN, and A-KI offered scientific and methodological considerations. All authors have read and approved the manuscript.

## Conflict of Interest

The authors declare that the research was conducted in the absence of any commercial or financial relationships that could be construed as a potential conflict of interest.

## Abbreviations

AKI: Anna-Karin Ivert
CNI: Care Need Index
CHC: Child Health Care
EM: Elisabeth Mangrio
ELN: Eva-Lotta Nilsson
LH: Lisa Hellström
MMR: Measles, Mumps, Rubella.
